# Social media–based interventions to prevent adolescent nicotine use: a scoping review

**DOI:** 10.1093/heapro/daag155

**Published:** 2026-10-21

**Authors:** Lisa Melber, Elena Engel, Sascha Gell, Jaroslava Kaňková, Raffael Heiss

**Affiliations:** Center for Social and Health Innovation, MCI Management Center Innsbruck, Innsbruck, Austria; Center for Social and Health Innovation, MCI Management Center Innsbruck, Innsbruck, Austria; Department of Communication, University of Vienna, Vienna, Austria; Center for Social and Health Innovation, MCI Management Center Innsbruck, Innsbruck, Austria; Department of Communication, University of Vienna, Vienna, Austria; Department of Communication, University of Vienna, Vienna, Austria; Center for Social and Health Innovation, MCI Management Center Innsbruck, Innsbruck, Austria

**Keywords:** adolescents, nicotine products, social media, health communication, scoping review

## Abstract

Despite growing concerns about adolescent nicotine use, the evidence regarding the outcomes of social media–based prevention efforts remains underexplored. This scoping review maps the scope, characteristics, and outcomes of (i) social media–based prevention interventions and (ii) educational interventions addressing adolescents’ interpretation of nicotine-related social media content. PubMed, PsycINFO, Scopus, ERIC, and CINAHL were systematically searched in March 2024 and updated in November 2025. Peer-reviewed English-language articles published since 2006 were included; cessation-focused studies were excluded. Of 1626 records screened by two independent reviewers, 18 studies met inclusion criteria. A structured codebook guided data extraction. The 18 included studies encompassed a wide range of designs, including online experiments, longitudinal and cross-sectional studies, content analyses, and qualitative research. Interventions ranged from message testing and simulated social media exposure to large-scale public health campaigns (e.g. “Rethink Vape,” “Truth,” “The Real Cost,” and “Fresh Empire”), alongside one school-based media literacy program (“MediaSense”). Most studies reported short-term improvements in knowledge, risk perception, or engagement, whereas evidence for sustained behavior change or reduced nicotine use was limited. Common limitations included reliance on self-reported outcomes and short follow-up periods. Most studies reported improvements in knowledge and risk perceptions, whereas evidence for sustained behavioral outcomes remains methodologically heterogeneous and largely focused on short-term outcomes. More rigorous longitudinal evaluations and sustained public health investment are needed. For practitioners and policymakers, the findings support investment in culturally tailored, platform-native campaigns alongside stronger regulatory frameworks to limit adolescents’ exposure to nicotine-related marketing in digital environments.

Contribution to Health Promotion

- Social media is an accessible, engaging channel for health promotion and nicotine prevention messaging aimed at adolescents and young people.
- This review shows that social media–based interventions may increase nicotine-related knowledge and risk awareness among young people aged 13–25 years.
- The evidence on changing nicotine use behavior and preventing initiation remains inconsistent and requires further investigation.
- Campaign characteristics such as influencer involvement, cultural tailoring, and interactive content may influence engagement and message retention among young audiences.
- Findings highlight the potential of social media as a health promotion tool and provide insights for optimizing campaign design, evaluation, and regulation.

## Introduction

Despite a global decline in tobacco consumption, the growing popularity of alternative nicotine products—such as e-cigarettes, snus, and nicotine pouches—has emerged as a pressing public health concern, particularly among adolescents (ESPAD Group 2020, Jackson *et al.* 2024, Tattan-Birch *et al.* 2024, World Health Organization 2024, Hammond and Reid 2025). Throughout this review, the term “nicotine products” refers to combustible tobacco products (e.g. cigarettes) and alternative nicotine products, including e-cigarettes, smokeless tobacco, snus, and nicotine pouches. E-cigarettes emit aerosols containing nicotine and other harmful substances, posing health risks not only to users but also to bystanders (Lietzmann and Moulac 2023). Smokeless products such as snus and nicotine pouches are also frequently marketed as safer alternatives. Snus is an oral smokeless tobacco product containing nicotine, whereas nicotine pouches contain nicotine but no tobacco and are available in a range of nicotine concentrations. Nicotine pouches in particular are increasingly marketed as alternatives to cigarettes and other tobacco products. They are often flavored and promoted within a harm-reduction framework, features that may enhance their appeal to youth (Dubrosa *et al.* 2023).

Social media plays a central role in the promotion of nicotine products, with companies employing targeted advertising, influencer endorsements, and viral marketing strategies to reach adolescent audiences (Guo *et al.* 2020). Social media platforms are deeply embedded in adolescents’ daily lives: ∼9 in 10 US teens use YouTube, at least 6 in 10 use TikTok or Instagram, and 36% report using at least one major platform almost constantly (Faverio and Sidoti 2025). Given this high level of engagement, adolescents may be particularly vulnerable to nicotine-related marketing tactics. The convergence of youth-oriented product design and aggressive digital promotion contributes to early nicotine initiation and dependence, heightening associated health risks (Overbeek *et al.* 2020, Lietzmann and Moulac 2023).

Against this backdrop, educational interventions that aim to strengthen adolescents’ media and health literacy may play an important role in enabling more critical evaluation of nicotine-related content on social media. At the same time, social media also holds significant potential as a vehicle for health education. These platforms can be leveraged to inform adolescents about the risks of nicotine use and to counter industry narratives (Cavazos-Rehg *et al.* 2021). However, the evidence regarding the outcomes of such prevention efforts remains underexplored, leaving a critical gap in both research and public health practice. A clearer understanding of what works—and under what conditions—is urgently needed to inform evidence-based responses to rising nicotine use among youth.

This study addresses this gap by conducting a scoping review of social media–based interventions aimed at preventing adolescent nicotine use, as well as educational interventions that specifically target adolescents’ exposure to and interpretation of nicotine-related marketing on social media. Following the Preferred Reporting Items for Systematic Reviews and Meta-Analyses extension for Scoping Reviews (PRISMA-ScR) guidelines (Tricco *et al.* 2018), the review systematically maps the characteristics and evaluated outcomes of these interventions and identifies key gaps in the existing evidence base. The findings aim to inform the development and refinement of future interventions and contribute to ongoing policy discussions on how best to protect adolescents from nicotine-related harms.

Accordingly, this scoping review aims to map the existing literature on social media–based nicotine prevention efforts targeting adolescents. Specifically, we examine (i) the types of interventions and campaigns studied, (ii) the social media platforms and delivery formats used, (iii) the study designs and methodological approaches applied, and (iv) the outcomes assessed. By systematically describing these characteristics, the review identifies methodological patterns and gaps in the current evidence base, including limitations related to outcome measurement, follow-up duration, and population coverage.

## Materials and methods

### Research design

This study employs a scoping review methodology to identify and synthesize existing research on social media–based interventions for adolescent nicotine prevention. A scoping approach was chosen because the field comprises diverse intervention types, study designs, and outcome measures, and the primary aim was to characterize the evidence base and identify research gaps rather than formally synthesize intervention effects. The review follows the PRISMA-ScR guidelines (Tricco *et al.* 2018). The protocol was preregistered with the Open Science Framework (OSF). The study protocol and the data extraction file are publicly available at https://osf.io/94u8n/?view_only=c5d4127117134bcaaf4e2151e08365f6.

### Search Strategy

A comprehensive search strategy was developed based on existing literature and key terms related to adolescents, nicotine use, social media, and educational or preventive interventions. An initial search string was discussed within the research team and iteratively refined before the final search strategy was agreed upon and implemented. The final search string was implemented across five databases: PubMed, PsycINFO, CINAHL, Scopus, and ERIC. The full search string is provided in Supplementary Annex A. The search was conducted in March 2024 using Boolean operators to combine keywords, explicitly excluding “cessation” to focus on preventive and educational measures only.

Eligibility was restricted to peer-reviewed research articles published in English, including primary empirical studies and evidence syntheses of empirical research. Although adolescence was the primary focus of the review, studies involving broader youth samples, including young adults up to age 25, were eligible when adolescents formed part of the study population. Eligible studies either evaluated prevention interventions delivered via social media platforms (e.g. campaigns, advertisements, or influencer-based content) or examined preventive educational interventions addressing adolescents’ exposure to and interpretation of nicotine-related social media and digital marketing content (e.g. media literacy approaches). Because social media constituted a core concept in the search strategy, records had to explicitly reference social media, digital media, or a named platform in the title or abstract to be retrieved.

Studies were excluded if they were nonresearch publication types (e.g. editorials, commentaries, viewpoints, or protocols), focused exclusively on populations outside the eligible age range, did not address an eligible social media or educational intervention, or primarily addressed nicotine cessation. Prevention and cessation interventions were distinguished because they differ substantially in their objectives, target populations, and intervention strategies. The publication period was restricted to studies published from 2006 onward to align with the emergence of major social media platforms such as Facebook, YouTube, and Twitter.

A two-step screening process was conducted using Rayyan reference management software (Ouzzani *et al.* 2016). First, duplicate studies were removed. Next, two reviewers independently screened the titles and abstracts based on predefined inclusion and exclusion criteria. Any disagreements were resolved through discussion or consultation with a third reviewer, ensuring consistency in study selection. Figure 1 outlines the full review process.

**Figure 1:**
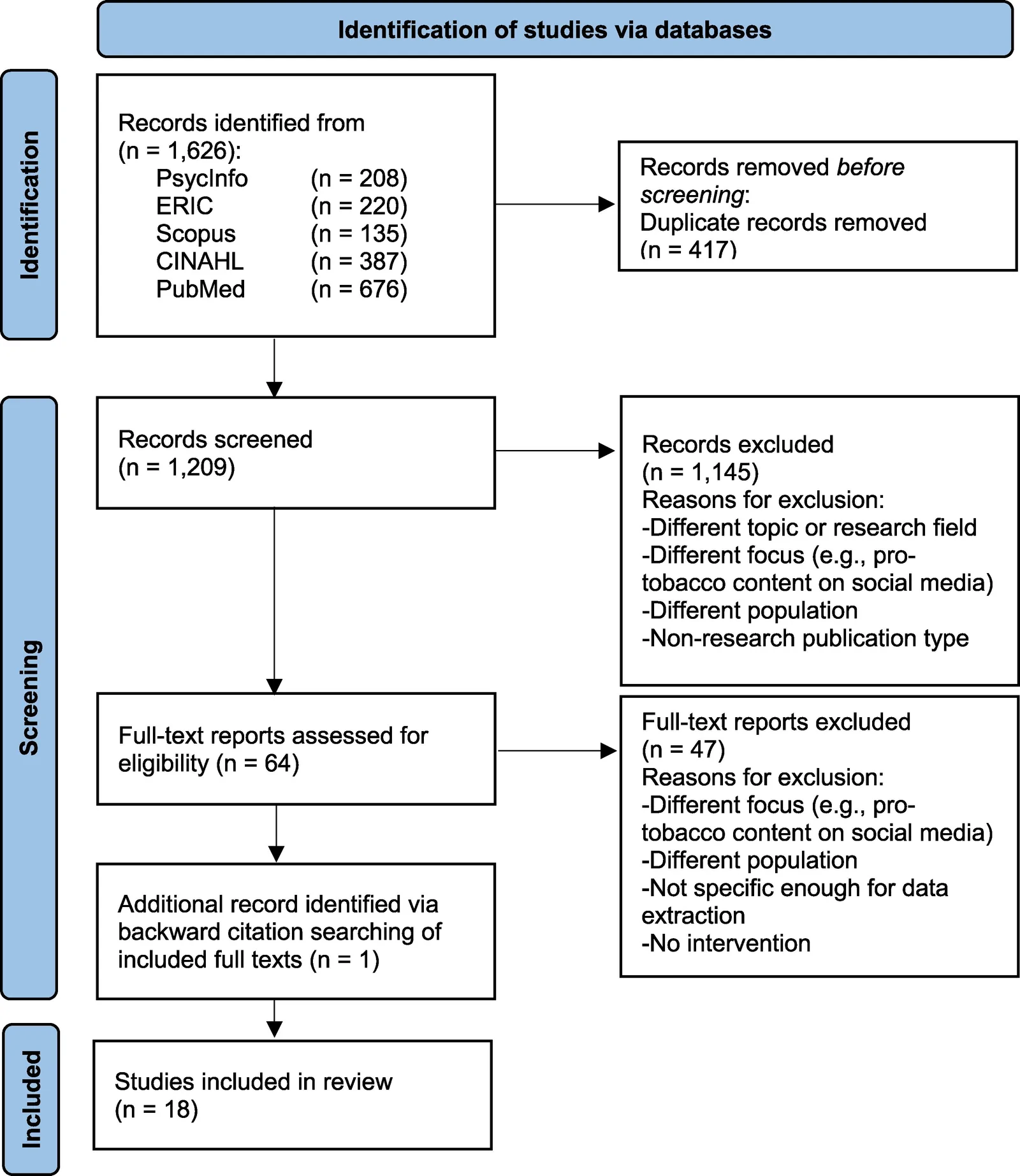
PRISMA-ScR flow diagram of study selection.

### Data Analysis

Following full-text review, 18 studies met the inclusion criteria and were selected for in-depth analysis. Data extraction was standardized using a predefined codebook, capturing key variables such as study design, target audience, social media platform, intervention components, and measured outcomes (Supplementary Annex B, see OSF for the full data extraction file). Additionally, campaign characteristics—including the use of influencers, interactive content, and platform-specific engagement strategies—were analyzed.

These variables were selected to capture how social media–based nicotine prevention efforts have been conceptualized and evaluated in the literature and to allow comparison across heterogeneous study designs. Mapping platforms, intervention features, and outcome types enabled the identification of methodological concentrations and gaps, particularly with respect to outcome measurement and the assessment of behavioral versus cognitive effects.

Studies were categorized according to the predefined dimensions of the data extraction framework, including sample characteristics, study design and methodology, social media platform, intervention characteristics, outcomes assessed, and key findings. Reported benefits, challenges, and recommendations for research and practice were subsequently grouped thematically across studies. Given the heterogeneity of study designs and outcomes, findings were synthesized narratively across these dimensions and themes. Ethical approval was not required because this study is a scoping review of published literature and did not involve human participants or identifiable personal data.

## Results

### Overview of studies

A total of 18 studies were included, encompassing a wide range of methodological approaches. These comprised randomized online experiments (*n* = 5), longitudinal observational studies (*n* = 3), a cross-sectional survey (*n* = 1), quantitative content analyses (*n* = 2), one observational program evaluation, one mixed-methods exploratory study, one participatory case study, one discrete choice experiment (DCE), one qualitative interview study, one prospective single-arm pre–post school-based media literacy intervention, and one systematic review. Content analyses were included only when they examined the delivery, reach, or engagement (e.g. user reactions) of prevention campaigns. This methodological diversity reflects the emerging and multidisciplinary nature of research on social media–based nicotine prevention. Table 1 provides an overview of the included studies.

**Table 1:** Characteristics, interventions, and key findings of the studies included in the scoping review.

Author(s), year	Study design	Nicotine product(s)	Study population	Intervention	Platform(s)	Outcomes measured	Major findings
Bottorff *et al*. (2014)	Exploratory descriptive mixed-methods study	Smoking	*N* = 135 adolescents; age 11–19 (grade 6–12); 54% female; aboriginal youth from across the province plus two high-school classrooms; Canada	Two brief (∼2 min), gender-specific YouTube-style videos linking tobacco/e-cigarette smoke exposure to breast cancer risk	YouTube (video format; not deployed on a live platform)	Reactions, risk perception, willingness to share, new knowledge gained	Videos rated favorably by both genders (avg. 4.5–4.6/5); >70% learned something new; ∼80% willing to share on social media
England *et al*. (2021)	Randomized online experiment	E-cigarettes	*N* = 268 adolescents; age 11–19; 55% female; mixed race/ethnicity (White, Hispanic, Black, Asian, American Indian/Alaska Native); USA	Rethink Vape campaign—risk-communication messages co-developed with adolescents; piloted before a 6-week launch	YouTube, Snapchat	Knowledge, risk perception, susceptibility, vaping intentions; postlaunch engagement	Increased knowledge, perceived risk, and intentions not to vape vs. control; campaign launch generated 3.8 million impressions and 770 000 completed video views
Evans *et al*. (2024)	Randomized dose–response experiment	E-cigarettes	*N* = 675 (478 never-users, 197 former users); age 18–24; USA	60-day antivaping social media intervention with four exposure-dose arms plus a no-exposure control	Facebook, Instagram	Future vaping intentions, anti-industry beliefs	Dose-related trend in beliefs but no significant reduction in future vaping intentions in either user group
Guo *et al*. (2020)	Observational program evaluation	Tobacco	Not reported; campaign-targeted hip-hop-identified youth aged 12–17, predominantly Black, Hispanic, and Asian American/Pacific Islander; USA	Fresh Empire campaign—microinfluencer content and paid digital/social media advertisements	Facebook, Instagram	Impressions, engagement, reach, sentiment, video completion	Microinfluencer and paid-ad content substantially exceeded impression targets, with predominantly positive audience sentiment (∼20 million impressions)
Karletsos *et al*. (2021)	Longitudinal survey (mediation analysis)	Smoking	N = 5069 adolescents; age 13–16; ∼48% male; urban Ghana	Combined mass media and social media antismoking/tobacco-control messaging	Twitter, Facebook, Instagram, YouTube, Snapchat	Smoking/shisha risk perception; mediation via peer and adult communication	Social media exposure increased shisha risk perception by 8.6 percentage points, partly mediated by peer (not adult) conversation
Kostygina *et al*. (2020)	Quantitative content analysis	Mixed	Campaign content: 308 216 tweets from 225 912 users (Truth campaign; designed for youth/young adults); USA	Truth campaign Twitter content—memes, hashtags, influencer posts	Twitter	Tweet volume/reach, content type, source impact, message valence, engagement	Influencer-driven, branded, shareable tweets achieved far higher reach and engagement; sentiment grew more positive over time (oppositional tweets fell from 25% to 5%)
Lazard (2021)	Randomized online experiment	E-cigarettes	*N* = 928 adolescents; age 15–18; ∼52% female; predominantly White, non-Hispanic; USA	Message-format testing (visual, quiz, text-only vs. no-message control) using simulated Instagram posts/stories	Simulated Instagram interface	Knowledge and beliefs about e-cigarette harms, message reactions, sharing preferences	All message formats increased knowledge and harm beliefs vs. control; most participants said they would share the messages, mostly with friends
Lin *et al*. (2023)	Quantitative content analysis	Mixed	Campaign content: 3515 posts and 28 125 comments from seven national/local antitobacco campaigns; USA	Antismoking information dissemination across multiple Facebook-based campaigns	Facebook	User engagement (likes, shares, comments), sentiment, content topic	Larger government/NGO campaigns had higher engagement; negative framing and video content increased shares
MacMonegle *et al*. (2024)	Longitudinal cohort study	E-cigarettes	*N* = 2625 US youth; age 11–16 at baseline	The Real Cost e-cigarette prevention campaign (digital/social media ads)	Facebook, Instagram, Snapchat	Campaign-targeted e-cigarette beliefs over time	Exposure to five of six ads increased targeted anti-e-cigarette beliefs (e.g. toxic metals, lung damage, and addiction risk)
Mylocopos *et al*. (2024)	Systematic review	E-cigarettes	39 studies reviewed (3 focused on social media); original participants generally <21 years	Synthesis of individual, school, and community/digital interventions to prevent e-cigarette initiation	Not specified (spans multiple campaigns/platforms)	Vaping initiation/prevalence, intentions, knowledge/attitudes, combustible tobacco use	Branded campaigns (The Real Cost, Rethink Vape, and Truth) improved knowledge/attitudes but showed inconsistent effects on vaping initiation
Van Den Heerik *et al*. (2017)	Participatory case study	Smoking	*N* = 441 slogans analyzed; target audience age 15–25; the Netherlands	Participatory co-created Dutch antismoking campaign (“Smoking is sóóó…”) using celebrity endorsements and audience-generated slogans	Facebook, Twitter	Valence, rhetorical style, and thematic content of co-created slogans	Audience-generated slogans matched campaign tone more closely on Twitter than Facebook, retaining negative valence toward smoking
Vereen *et al*. (2022)	Cross-sectional survey	E-cigarettes	*N* = 623 US adolescents (nationally representative); age 13–17	The Real Cost e-cigarette prevention ads (aided-recall evaluation)	Multiple (e.g. YouTube, Facebook, Instagram)	Aided recall of prevention/pro-vaping ads, social media use, demographics	71% recalled at least one ad; recall was significantly higher among Black participants and medium/high social media users
Lazard *et al*. (2025)	Randomized online experiment	E-cigarettes	*N* = 712 young adults; age 18–25; USA	Antivaping messages emphasizing health harms alone vs. health harms + social impact, vs. control	Simulated social media environment (“Clickbrite”)	Sharing intentions, PME, relevance, knowledge, and beliefs	Harm-focused messages increased sharing intentions and discouraged vaping; sharing occurred mainly via private/ephemeral channels
Pluta *et al*. (2025)	DCE	E-cigarettes	*N* = 245 sexual and gender minority (SGM/LGBTQ+) youth; age 13–18; USA	36 Instagram-style images varying in SGM tailoring level (none/low/high) and number of people depicted	Instagram	Stated preferences for, attention to, and sharing likelihood of images	Images with high tailoring and two people were most preferred and most likely to be shared
Henes *et al*. (2025)	Longitudinal study	Tobacco	*N* = 5129 individuals (10 674 survey responses); age 12–18; hip-hop-identified youth; USA	Fresh Empire campaign—awareness and receptivity evaluation	YouTube, Facebook, Instagram	Brand/ad awareness, perceived effectiveness	Greater hip-hop identification and digital exposure predicted higher ad awareness; perceived effectiveness was lower among smokers/susceptible youth
Leung *et al*. (2025)	Randomized online experiment	E-cigarettes	*N* = 614 young people; age 16–25; Australia	Youth-codesigned generative-AI vaping-awareness ads vs. existing agency ads, with/without AI source labeling	Instagram, Facebook	PME across five measures	AI-generated ads were noninferior to agency ads and scored slightly higher on most effectiveness measures
Michaud *et al*. (2025)	Prospective single-arm pre–post study	E-cigarettes	*N* = 384 middle/high-school students; age 11–18; five schools (Nebraska/Iowa, USA)	MediaSense school-based media literacy curriculum (nine modules, ∼20 min each)	Classroom e-learning (not delivered via a live social media platform)	Vaping media literacy, harm perception, knowledge, susceptibility to vaping	Significant gains in media literacy and knowledge; susceptibility to vaping was significantly reduced
Theis *et al*. (2025)	Qualitative interview study	E-cigarettes	*N* = 34 (29 interviews, 5 paired); SGM/LGBTQ + youth; age 13–17; USA	Semi-structured interviews reflecting on four existing antivaping social media messages	TikTok, Instagram, YouTube	Perceived effectiveness, credibility, representation, cultural tailoring	Authentic representation and an affirming tone were valued most; peer/LGBTQ + creators seen as more credible than institutional sources

NGO, nongovernmental organizations; PME, perceived message effectiveness.

### Methodological assessment

The included methods were diverse and provide different levels of causal information. Five articles reported randomized online experiments, which were small to medium in size and delivered entirely online, with automated assignment and message delivery. Although randomized designs offer the most robust causal evidence, the experimental studies included in this review are characterized by short observation periods and, in some cases, high loss to follow-up, limiting conclusions about sustained behavioral outcomes (England *et al.* 2021, Lazard 2021, Evans *et al.* 2024, Lazard *et al.* 2025, Leung *et al.* 2025). A full quality appraisal of experiments included is provided in Supplementary Annex C. In addition, one study employed a DCE to examine stated preferences and trade-offs related to vaping prevention messages, providing experimental evidence on choices rather than observed behavior (Pluta *et al.* 2025).

Two studies include longitudinal data with large and representative samples (Karletsos *et al.* 2021, MacMonegle *et al.* 2024).

One study used a cross-sectional survey design, namely the national aided-recall survey (Vereen *et al.* 2022). Bottorff *et al.* (2014) employed an exploratory descriptive mixed-methods design involving the development of researcher-produced videos followed by a youth evaluation combining questionnaire data and qualitative feedback. Both studies relied substantially on self-reported outcomes. Vereen *et al.* (2022) may therefore be subject to recall and unmeasured confounding, whereas the convenience sample and descriptive design used by Bottorff *et al.* (2014) limit generalizability and do not permit conclusions about intervention effectiveness. One study used a prospective, interventional, single-arm pre–post design across five schools (Michaud *et al.* 2025).

The two content analyses (Kostygina *et al.* 2020, Lin *et al.* 2023) combined human and machine coding, using publicly available data from Twitter or Facebook. These studies provide valuable insights into campaign content and engagement patterns but do not allow inference about individual-level exposure or behavioral outcomes. Furthermore, one systematic review (Mylocopos *et al.* 2024) synthesizes evidence on youth e-cigarette prevention across broader intervention settings.

While the diversity of designs allowed for broad exploration of intervention strategies and campaign outcomes, comparability across studies was limited by differences in metrics, sample frames, and intervention intensity.

### Sample characteristics

All included studies targeted adolescents or youth audiences. Many studies included broader youth samples that also encompassed young adults (e.g. ages up to 24 or 25), reflecting the target audiences of several prevention campaigns, but most focused on youth between 13 and 19 years. The majority of studies were conducted in the USA (*n* = 14), with one study each from Canada, the Netherlands, and Australia, and only one study conducted in the Global South (Ghana). Several studies explicitly focused on or oversampled specific at-risk subgroups, including sexual and gender minority (SGM/LGBTQ+) youth (Pluta *et al.* 2025, Theis *et al.* 2025), adolescents identifying with hip-hop cultural peer crowds (Guo *et al.* 2020, Henes *et al.* 2025), and racially or socioeconomically diverse populations (Evans *et al.* 2024, MacMonegle *et al.* 2024). Reporting of socioeconomic characteristics, sexual identity, and household-level nicotine exposure was inconsistent across studies, limiting comparability. Only a small subset of studies reported detailed household-level variables, such as parental communication about smoking or the presence of nicotine users in the home.

### Platforms

Most interventions leveraged mainstream social media platforms, including Instagram, Facebook, YouTube, Twitter/X, and Snapchat, often as part of multi-platform campaign strategies (e.g. “Truth,” “The Real Cost,” and “Fresh Empire”). Several studies explicitly tailored content to platform-specific affordances, such as Instagram stories and visuals, Twitter/X hashtags, memes, and influencer amplification, or short-form video formats. A subset of experimental studies tested messages in simulated social media environments rather than live feeds (Lazard 2021, Lazard *et al.* 2025), allowing controlled exposure but limiting ecological validity. One study evaluated a school-based media literacy intervention (“MediaSense”) addressing adolescents’ interpretation of social media and digital marketing content rather than delivering messages directly via social media platforms (Michaud *et al.* 2025).

Influencer-led strategies were prominent in large-scale campaigns such as Truth and Fresh Empire, while other interventions relied on paid digital advertising, co-created content, or youth-designed messages, including generative artificial intelligence (AI)-assisted ads compared against official public health agency materials (Van Den Heerik *et al.* 2017, Leung *et al.* 2025). Although several studies reported engagement-related indicators (e.g. impressions, likes, shares, recall, or sharing intentions), measurement approaches varied widely, and few captured actual attention, exposure dose, or sustained engagement, limiting insights into dose–response relationships and platform-level performance.

### Measured outcomes

Measured outcomes varied widely across studies. Frequently assessed outcomes included knowledge of nicotine products (Bottorff *et al.* 2014, England *et al.* 2021, Karletsos *et al.* 2021, Lazard 2021, MacMonegle *et al.* 2024, Mylocopos *et al.* 2024, Lazard *et al.* 2025, Leung *et al.* 2025, Michaud *et al.* 2025) and attitudes toward nicotine products (Bottorff *et al.* 2014, England *et al.* 2021, Karletsos *et al.* 2021, Lazard 2021, Mylocopos *et al.* 2024, Leung *et al.* 2025, Theis *et al.* 2025). Many studies also assessed risk perceptions/harm beliefs and message evaluations (e.g. perceived effectiveness, relevance, and credibility), particularly in online experimental designs (England *et al.* 2021, Lazard 2021, Evans *et al.* 2024, Lazard *et al.* 2025, Leung *et al.* 2025). Behavioral precursors—such as susceptibility, intentions, and willingness to share messages—were common (England *et al.* 2021, Lazard 2021, Evans *et al.* 2024, Lazard *et al.* 2025, Michaud *et al.* 2025, Pluta *et al.* 2025). Measures of campaign exposure, awareness, and recall appeared in evaluations of real-world campaigns (Vereen *et al.* 2022, MacMonegle *et al.* 2024, Henes *et al.* 2025). Only a subset assessed nicotine/vaping use outcomes, typically via self-report rather than objective validation (England *et al.* 2021, Karletsos *et al.* 2021, Mylocopos *et al.* 2024, Lazard *et al.* 2025, Leung *et al.* 2025, Michaud *et al.* 2025, Theis *et al.* 2025). Engagement indicators (e.g. impressions, likes, shares/retweets, comments, and sentiment) were most prominent in campaign evaluations and content-analytic studies (Guo *et al.* 2020, Kostygina *et al.* 2020, Lin *et al.* 2023).

### Types of interventions and campaigns

Across the 18 included studies, interventions encompassed small-scale message testing, participatory and AI-supported co-creation, DCEs, and media literacy education (Bottorff *et al.* 2014, Lazard 2021, Leung *et al.* 2025, Michaud *et al.* 2025, Pluta *et al.* 2025). One exploratory study developed and pretested educational videos intended for social media dissemination but did not evaluate real-world platform delivery or behavioral outcomes (Bottorff *et al.* 2014). In addition, several studies examined professionally developed, large-scale public health campaigns evaluated in real-world settings, including “Rethink Vape,” Truth, The Real Cost, and Fresh Empire (Guo *et al.* 2020, Kostygina *et al.* 2020, England *et al.* 2021, Vereen *et al.* 2022, Evans *et al.* 2024, MacMonegle *et al.* 2024, Henes *et al.* 2025). Intervention intensity varied substantially, ranging from brief, one-time exposures or message mock-ups presented in experimental or simulated social media environments (Lazard 2021, Lazard *et al.* 2025, Leung *et al.* 2025) to longer-running campaigns assessed via single or repeated surveys of awareness, exposure, and belief change (MacMonegle *et al.* 2024, Henes *et al.* 2025). Smaller initiatives frequently emphasized participatory content co-creation and message tailoring, whereas large-scale campaigns were supported by substantial institutional funding and employed diverse strategies, including high-salience harm messaging, influencer collaborations, and culturally tailored branding. An overview of major real-world campaigns evaluated in the included studies is provided in Table 2.

**Table 2:** Real-world campaigns evaluated in the included studies, including their institutional sponsors, thematic focus, and communication strategies.

Campaign (studies)	Funding	Focus	Features
Rethink Vape (England *et al.* 2021, Mylocopos *et al.* 2024)	Virginia Foundation for Healthy Youth	Emphasizes risks of chemical compounds in e-cigarettes; detrimental health outcomes (e.g. respiratory damage)	Uses brief, visually compelling content; vibrant colors and bold typography
Truth (Kostygina *et al.* 2020, Lin *et al.* 2023, Mylocopos *et al.* 2024)	Truth Initiative (established via 1998 Master Settlement)	Addresses respiratory damage, stress, and anxiety; exposes deceptive marketing practices of tobacco and nicotine industries	Uses memes, branded hashtags, influencer posts; targets vulnerable communities
The Real Cost (Vereen *et al.* 2022, Lin *et al.* 2023, Mylocopos *et al.* 2024, MacMonegle *et al.* 2024)	US FDA	Focuses on metal particles, anxiety, bone density issues; illustrates emotional consequences (e.g. disappointing family)	Predominantly dark theme to convey gravity; uses fear-based imagery with adolescent subjects
Fresh Empire (Guo *et al.* 2020, Henes *et al.* 2025)	US FDA	Targets hip-hop culture youth; emphasizes tobacco industry manipulation and health risks	Uses culturally relevant messaging; leverages social media influencers and paid advertisements

FDA, Food and Drug Administration.

### Key findings on knowledge and behavior

Across the included studies, most interventions were associated with short-term improvements in knowledge, harm perceptions, and message evaluations, while evidence for sustained behavior change was limited. Online experiments showed increases in vaping-related knowledge, perceived harms, and perceived message effectiveness following exposure to prevention messages (England *et al.* 2021, Lazard 2021, Lazard *et al.* 2025, Leung *et al.* 2025). Effects were most consistently observed for outcomes closely tied to message processing (e.g. knowledge, perceived risk, relevance, and perceived effectiveness) rather than downstream behavioral outcomes.

Several studies suggested that greater campaign exposure may be linked to stronger prevention-relevant beliefs, although measurement approaches varied. In a randomized dose–response experiment, Evans *et al*. (2024) reported evidence consistent with exposure effects on vaping-related beliefs but did not find a significant reduction in future vaping intentions, particularly among nonusers. A longitudinal cohort evaluation of The Real Cost found that campaign exposure was associated with increases in campaign-targeted beliefs over time (MacMonegle *et al.* 2024). In Ghana, Karletsos *et al*. (2021) reported that exposure to tobacco-control messaging—including social media exposure—was associated with higher smoking risk perceptions, partly mediated by peer communication.

Evidence for effects on behavioral precursors was more common than evidence on actual use. Lazard *et al*. (2025) found that harm-focused antivaping messages increased intentions to share content and improved perceived message effectiveness and related cognitive outcomes, with sharing most often reported via private or ephemeral channels. Michaud *et al*. (2025) reported that a school-based media literacy intervention (MediaSense) improved vaping media literacy and harm perceptions and was associated with reduced susceptibility to vaping in a pre–post design, suggesting a plausible pathway through literacy and risk appraisal.

Qualitative and preference-based studies highlighted that “what works” may differ across subgroups and contexts. Sexual and gender minority youth often emphasized credibility, representation, and an affirming tone and described preferences for messages perceived as authentic and nonstigmatizing (Theis *et al.* 2025). Pluta *et al*. (2025) further showed that tailoring and representation shape message preferences among SGM youth, underscoring the importance of audience segmentation for campaign design. Content-analytic and campaign evaluation studies also emphasized the role of influencers, memes, and platform-native formats in driving reach and engagement (Guo *et al.* 2020, Kostygina *et al.* 2020, Lin *et al.* 2023), although engagement metrics were heterogeneous and rarely linked to long-term outcomes.

Overall, while most studies documented short-term improvements in knowledge, attitudes, beliefs, and intentions, evidence for sustained behavior change or reduced nicotine use remains limited. The existing systematic review addressed youth vaping prevention across broader settings (not limited to social media) and similarly concluded that branded prevention campaigns (including Rethink Vape, Truth, and The Real Cost) improved knowledge and risk perceptions but did not demonstrate clear prevention of vaping initiation (Mylocopos *et al.* 2024).

### Identified benefits

One of the foremost advantages of social media campaigns identified by the included studies is their capacity to reach vast and diverse audiences, effectively engaging specific cultural groups and niche audiences. Because social media platforms enable highly tailored messaging, campaigns can address the varied preferences and needs of young people, including priority subgroups such as sexual and gender minority youth (Bottorff *et al.* 2014, Guo *et al.* 2020, Karletsos *et al.* 2021, Henes *et al.* 2025, Pluta *et al.* 2025, Theis *et al.* 2025). Additionally, real-time feedback mechanisms—such as likes, shares, and comments—allow interventions to be adjusted and scaled at relatively low cost, making social media more cost-effective than traditional media (Guo *et al.* 2020, Kostygina *et al.* 2020).

Another key benefit is the use of engaging formats, notably videos and influencer partnerships, which resonate strongly with adolescents by increasing credibility and authenticity. Influencers who communicate in their own voice help make health messages more relatable and trustworthy to young audiences (Guo *et al.* 2020, Henes *et al.* 2025, Theis *et al.* 2025). Meanwhile, tailored interactive content—such as quizzes or polls—fosters active participation, leading to improved retention and engagement (Kostygina *et al.* 2020). Leung *et al*. (2025) further demonstrated the feasibility and speed of generative AI with youth codesign to produce effective social media ads, with noninferiority and small advantages over existing agency content.

Finally, the organic dissemination of campaign material among peers emerged as a significant advantage. Young people frequently share these materials within their own social circles, leveraging peer dynamics to reinforce nonsmoking norms. This peer-driven dissemination extends a campaign’s reach well beyond its initial audience, thereby amplifying impact and promoting healthier behaviors among young people (Guo *et al.* 2020, England *et al.* 2021, Lazard *et al.* 2025).

These affordances make social media particularly well-suited for rapid, scalable, and culturally responsive prevention efforts targeting youth across demographic boundaries.

### Identified challenges

A central obstacle noted by several studies is the pervasive presence of pro-nicotine content and targeted tobacco industry advertising, which directly competes with public health messages (Bottorff *et al.* 2014, Kostygina *et al.* 2020, England *et al.* 2021, Lazard 2021, MacMonegle *et al.* 2024, Lazard *et al.* 2025). This includes influencer marketing and paid promotions by the tobacco and e-cigarette industries, creating a competitive digital environment where public health campaigns must contend with high-volume, persuasive content that glamorizes nicotine use. At the same time, adolescents may struggle to discern credible information sources—particularly in spaces where influencer and user-generated content often carries equal or greater perceived authority than institutional sources. This makes it difficult for public health campaigns to stand out in a landscape saturated with misleading or promotional vaping content (England *et al.* 2021, Lazard 2021, Michaud *et al.* 2025). Henes *et al*. (2025) further highlighted uncertainty about whether adolescents meaningfully engage with campaign content, as existing metrics cannot determine whether messages are viewed in full or receive sustained attention.

Moreover, campaigns faced challenges related to sustained implementation, including the financial and logistical burden of running high-frequency, long-duration campaigns (Kostygina *et al.* 2020, Lin *et al.* 2023, Evans *et al.* 2024). While social media offers cost advantages over traditional media, meaningful engagement still requires investment in content creation, ad placement, and ongoing monitoring (Guo *et al.* 2020, England *et al.* 2021). Campaigns must also navigate the rapid evolution of social media platforms, the rise of AI technologies, and the diversity in young people’s literacy levels, access to technology, and content preferences (Guo *et al.* 2020, Kostygina *et al.* 2020, Henes *et al.* 2025, Leung *et al.* 2025, Michaud *et al.* 2025, Pluta *et al.* 2025). Thus, campaigns must continually adapt their formats, styles, and messaging tone to remain relevant.

Finally, influencer communication emerged as a double-edged sword. While it can expand reach and increase credibility, it also introduces risks to message consistency and authenticity. If influencers appear overly scripted, stray from core health messages, or endorse conflicting content, the campaign’s credibility may suffer (Guo *et al.* 2020, Kostygina *et al.* 2020, Theis *et al.* 2025). Additionally, monitoring influencer posts for alignment with public health goals requires dedicated oversight, which may be difficult to sustain in resource-constrained settings (Guo *et al.* 2020, Kostygina *et al.* 2020).

## Discussion

This review identified a small body of literature evaluating social media–based interventions to prevent nicotine use among adolescents. Importantly, only one study evaluated an educational intervention explicitly designed to strengthen young people’s ability to critically interpret nicotine-related social media marketing and content on social media (Michaud *et al.* 2025). This highlights an important gap, as adolescents are increasingly embedded in highly commercialized social media environments, in which influencer-driven communication and algorithmic amplification blur the boundaries between marketing, persuasion, and credible health information (Heiss *et al.* 2025).

### Implications for research

The review identified several important gaps in the current evidence base. Although many studies reported improvements in awareness, knowledge, and risk perceptions, only a small number evaluated behavioral outcomes (England *et al.* 2021, Lazard 2021, MacMonegle *et al.* 2024, Michaud *et al.* 2025), and those that did often relied on self-reported measures or short follow-up periods (Evans *et al.* 2024, Mylocopos *et al.* 2024). Consequently, little is known about whether improvements in engagement, recall, or perceived effectiveness translate into sustained reductions in nicotine initiation or use.

Methodologically, the literature remains heterogeneous. Experimental studies provide valuable evidence regarding causal mechanisms but were frequently underpowered and limited to short observation periods (England *et al.* 2021, Lazard 2021, Evans *et al.* 2024, Leung *et al.* 2025). Content analyses and cross-sectional studies generated important insights into campaign characteristics and user engagement but could not establish intervention effectiveness (Kostygina *et al.* 2020, Vereen *et al.* 2022, Lin *et al.* 2023). Future research should prioritize adequately powered longitudinal and experimental evaluations, standardized outcome measures, and cross-platform assessments that reflect adolescents’ increasingly fragmented social media use. Greater attention should also be paid to educational interventions that strengthen media literacy and critical evaluation skills, an area that remains remarkably underrepresented, with only one identified study specifically evaluating a media literacy intervention targeting nicotine-related social media content (Michaud *et al.* 2025).

The existing evidence was also unevenly distributed across nicotine product categories. Most included studies focused on e-cigarette and vaping prevention, with some addressing cigarette smoking or broader tobacco prevention. We identified no intervention specifically targeting the prevention of nicotine pouch use, despite the growing popularity of these products among adolescents and young adults (ESPAD Group 2020, Hammond and Reid 2025). This imbalance suggests that prevention research has not yet kept pace with changes in the nicotine product landscape. Future studies should evaluate social media–based prevention strategies targeting nicotine pouches and other emerging nicotine products while examining whether interventions developed for cigarettes or e-cigarettes can be effectively adapted to these newer forms of nicotine use.

### Implications for practice

The reviewed interventions show that campaigns that incorporate influencer partnerships, culturally tailored messaging, youth participation, and interactive content may achieve greater engagement and message recall. These approaches likely resonate because they align with how adolescents communicate and learn—informally, socially, and through emotionally engaging content (Sumter *et al.* 2009, Engel *et al.* 2024, Theis *et al.* 2025).

At the same time, the review demonstrates that social media campaigns alone are unlikely to be sufficient. Prevention efforts should increasingly integrate media literacy approaches that equip adolescents to critically evaluate nicotine-related marketing and health information encountered on digital platforms. Campaigns should therefore be designed with adolescents rather than simply for adolescents, ensuring authenticity, cultural relevance, and adaptability to rapidly evolving social media environments.

### Implications for policy

The reviewed studies also highlight broader structural challenges. Public health campaigns continue to compete with extensive commercial promotion of nicotine products, with e-cigarette advertising expenditures alone reaching an estimated $859.4 million in 2021 (Federal Trade Commission 2024). This imbalance puts public health campaigns at a disadvantage, not only in reach but in visibility and frequency of exposure.

The findings support evidence-informed policy measures. This includes strengthening restrictions on paid tobacco promotion and influencer marketing, improving transparency through advertising disclosure requirements, and enhancing platform-level protections such as age-gating and content moderation (Kong *et al.* 2024). At the same time, governments and public health agencies should invest not only in campaign implementation but also in independent evaluation, platform-specific expertise, and sustainable media literacy initiatives. Such investments are needed to ensure that prevention efforts remain responsive to rapidly evolving digital communication environments while avoiding unintended consequences that could inadvertently reinforce access to more harmful products (Morphett *et al*. 2021, Banks *et al*. 2022).

### Limitations

This scoping review identified a small but diverse body of research on social media–based interventions for adolescent nicotine prevention. Strengths include a broad multi-database search, the inclusion of varied study designs, and attention to both intervention characteristics and outcomes. However, several limitations should be acknowledged. First, as a scoping review, the primary aim was to map the scope and characteristics of existing research rather than formally assess effectiveness or conduct a quantitative synthesis. Second, the review was limited to peer-reviewed English-language publications, which may have excluded relevant gray literature or unpublished campaign evaluations. In addition, because the search strategy required explicit reference to social media or specific social media platforms in searchable fields, some broader tobacco prevention campaign evaluations incorporating social media components may not have been identified. Rapid changes in social media platforms may also affect the longer-term generalizability of the findings as platform features, dominant formats, and algorithmic curation continue to evolve. Finally, the review was restricted to prevention-focused interventions and therefore does not capture evidence from cessation-focused campaigns. Future evidence syntheses could compare prevention and cessation interventions to identify shared and distinct social media strategies for reducing nicotine use among young people at different stages of behavior change.

## Conclusion

While social media campaigns show promise for nicotine prevention among adolescents, the existing evidence base remains limited and methodologically heterogeneous, complicating comparisons across studies. Given the continuing burden of adolescent nicotine use, evidence-informed regulation, sustained prevention efforts, and rigorous evaluation remain essential to reduce adolescents’ exposure to nicotine-related marketing while avoiding unintended consequences that could reinforce access to more harmful products (Morphett *et al*. 2021, Banks *et al*. 2022). Future social media–based nicotine prevention efforts should combine culturally tailored messaging, media literacy, and rigorous longitudinal evaluation to maximize their public health impact.

## Supplementary Material

daag155_Supplementary_Data

## Data Availability

The study protocol and complete data extraction file underlying this review are publicly available via the Open Science Framework (OSF) at https://osf.io/94u8n/?view_only=c5d4127117134bcaaf4e2151e08365f6.
