# Supplementary material for: Social media–based interventions to prevent adolescent nicotine use: a scoping review
Source: Health Promot Int. 2026 Oct 21;41(5):daag155. doi: 10.1093/heapro/daag155 (PMC13684276; doi:10.1093/heapro/daag155)
Supplement: Supplementary file 1 [file daag155_supplementary_data.docx]

**Supplementary Material**

**Annex A. Search String (Example for PubMed*)***

("youth"[Title/Abstract] OR "young"[Title/Abstract] OR "teen*"[Title/Abstract] OR "adolescent") AND ("tobacco"[Title/Abstract] OR "snus"[Title/Abstract] OR "pouch"[Title/Abstract] OR "nicotine"[Title/Abstract] OR "cigarette"[Title/Abstract] OR "vaping"[Title/Abstract] OR "nicotine delivery system"[Title/Abstract] OR "e-cigarette"[Title/Abstract] OR "electronic cigarette"[Title/Abstract] OR "ENDS"[Title/Abstract] OR "vape"[Title/Abstract] OR "smoking") AND ("health education"[Title/Abstract] OR "health promotion"[Title/Abstract] OR "prevention"[Title/Abstract] OR "education"[Title/Abstract] OR "campaign"[Title/Abstract] OR "intervention"[Title/Abstract] OR "strategy*"[Title/Abstract] OR "literacy"[Title/Abstract] OR "awareness"[Title/Abstract] OR "initiative"[Title/Abstract] OR "anti-tobacco") AND ("social media"[Title/Abstract] OR "Facebook"[Title/Abstract] OR "Instagram"[Title/Abstract] OR "TikTok"[Title/Abstract] OR "digital media"[Title/Abstract] OR "Internet"[Title/Abstract] OR "influencer"[Title/Abstract] OR "YouTube"[Title/Abstract] OR "Twitter"[Title/Abstract] OR "Snapchat"[Title/Abstract] OR "VK"[Title/Abstract] OR "Weibo"[Title/Abstract] OR "blog*"[Title/Abstract] OR "vlog*"[Title/Abstract] OR "WeChat"[Title/Abstract] OR "QQ"[Title/Abstract] OR "Qzone"[Title/Abstract] OR "Douyin"[Title/Abstract] OR "Kuaishou"[Title/Abstract] OR "Baidu Tieba"[Title/Abstract] OR "Discord"[Title/Abstract] OR "Twitch"[Title/Abstract] OR "LinkedIn"[Title/Abstract] OR "Xing"[Title/Abstract] OR "WhatsApp"[Title/Abstract] OR "Reddit"[Title/Abstract] OR "Signal"[Title/Abstract] OR "Telegram"[Title/Abstract] OR "Pinterest"[Title/Abstract] OR "MySpace"[Title/Abstract] OR "Tumblr*"[Title/Abstract] OR "Kik"[Title/Abstract] OR "iMessage"[Title/Abstract] OR "SMS"[Title/Abstract] OR "Vine"[Title/Abstract] OR "forum*"[Title/Abstract] OR "podcast*"[Title/Abstract] OR "livestream*"[Title/Abstract] OR "reels"[Title/Abstract]) NOT "cessation"[Title/Abstract]

**Annex B. Codebook for data extraction**

| **Basic study informatio**n | |
| --- | --- |
| Authors | Name of the study authors |
| Year of publication | Year when the study was published |
| Study design | Type of the study design (for reviews: number of studies included) |
| Journal; publisher | Name of the journal and publisher |
| Focus of the study | Main focus or topic of the study |
| Objective, research question, and hypothesis | the study’s objective, research question, and hypothesis |
| **Social Media Platform** | |
| Used social media platform | the social media platform(s) used in the study |
| Sample details |  |
| Sample size | Number of participants or objectives (e.g. posts) |
| Age range | Age range of participants |
| Demographic information | Key demographic details of participants (e.g. gender, ethnicity) |
| Other characteristics | additional characteristics of participants (e.g. education level) |
| **Intervention details** | |
| Intervention strategy | Description of the strategy or approach used (e.g. videos, influencer collaboration) |
| Name of intervention | Name given to the intervention or campaign |
| Place of intervention | Location where the intervention was implemented |
| Duration of intervention | Length of time the intervention was conducted |
| **Outcomes measure** | |
| Primary outcome measure | Detailed description of the main outcomes measured in the study |
| **Main results** | |
| Key findings | Summary of results or findings |
| **Challenges in implementing and using social media for educational interventions** | |
| Challenges identified through study data | Specific challenges identified in the study data |
| Additional challenges mentioned by the authors | Other challenges noted by the authors |
| **Benefits of using social media for educational interventions** | |
| Benefits identified through study data | Specific benefits identified in the study data |
| Additional benefits mentioned by the authors | Other benefits noted by the authors |
| **Study limitations** | |
| Limitations | Limitations specific to the study |
| **Recommendations and future directions** | |
| For practice | suggestions for improving social media-based educational interventions |
| For research | directions for future research on this topic |

**Annex C. Quality Assessment of Randomized Controlled Studies**

Five studies included in this review employed randomized designs (England et al., 2021; Evans et al., 2024; Lazard, 2020; Lazard et al., 2025; Leung et al., 2025) and were assessed using established quality criteria for randomized trials (Higgins et al., 2019; Tufanaru et al., 2020). All five studies applied random allocation, used appropriate statistical analyses, and clearly defined outcome measures. None of the studies reported allocation concealment, and blinding of participants, intervention providers, or outcome assessors was not formally addressed. However, the fully automated digital delivery of interventions via online platforms – where participants only received content and survey items tailored to their assigned group – likely reduced the risk of contamination and group awareness. Nevertheless, participants could still infer their assignment (e.g., receiving anti-vaping messages vs. no message), which limits full participant blinding and introduces a residual risk of performance bias. Additionally, all studies relied exclusively on self-reported outcomes, which may be subject to detection bias, particularly in the absence of objective or blinded assessments.

Two studies (Evans et al., 2024; Leung et al.,2025) reported a priori power analysis. Leung et al. (2025) reported achieving over 95% power for its noninferiority hypothesis and 80% power for its labeling-effects hypothesis, based on a preregistered sample size calculation. While all five studies found statistically significant or noninferior effects on key outcomes such as knowledge, beliefs, sharing intentions, and perceived message effectiveness, the absence of reported power calculations in the other three studies raises concerns about their ability to detect more nuanced effects, including subgroup differences or interactions - a particular concern for Lazard et al. (2025), which examined several race, ethnicity, and vaping-status subgroup comparisons without a priori justification for adequate power to detect interaction effects.

Manipulation or treatment checks were largely absent across the reviewed studies, with one notable exception: Leung et al. (2025) directly assessed whether participants correctly identified their assigned source-labeling condition and conducted sensitivity analyses restricted to participants who passed this check, strengthening confidence in the validity of its labeling-effect findings. Lazard et al. (2025) also recorded behavioral exposure and interaction data (e.g., scroll depth, likes, comments) within its simulated social media feed, which functions as an informal treatment-engagement check, though it does not confirm participants attended to or comprehended message content in the way a formal manipulation check would. The remaining studies did not report any manipulation or treatment checks, making it unclear whether participants in those trials engaged with the intervention content as intended.

Confounding factors were not systematically addressed across the studies. Evans et al. (2024) adjusted for basic demographic variables, and Leung et al. (2025) used mixed-effects models accounting for repeated measures within participants and ads while adjusting for message theme; Lazard et al. (2025) examined race, ethnicity, and vaping status as moderators but did not adjust for baseline attitudes or peer influences. None of the five studies accounted for potentially influential factors such as prior message exposure, baseline attitudes toward vaping, or peer influence, which limits internal validity and interpretability across the evidence base.

Ecological validity varied across the designs. Lazard (2020) and Lazard et al. (2025) both employed a lab-like mock social media interface (clikbrite, based on the Truman Platform; Ruo et al., 2020; DiFranzo et al., 2018) that allowed for high experimental control but may limit generalizability, as adolescents' and young adults' engagement with content in real-world social media environments could differ from a simulated, researcher-controlled feed. Leung et al. (2025) instead presented ads as static stimuli without an embedded social feed, but drew comparison materials directly from existing official health agency campaigns, which may improve external validity for that specific comparison while introducing potential selection bias in which existing ads were chosen.

Only one study was formally pre-registered with a published protocol: Leung et al. (2025) was registered on ClinicalTrials.gov (NCT07042789) and had its hypotheses and design preregistered via OSF, representing a meaningful improvement in transparency relative to the other four studies, none of which reported pre-registration or a detailed a priori protocol.

In summary, while the included randomized studies demonstrate promising effects and make appropriate use of digital intervention delivery, randomized controlled trials remain the gold standard for evaluating intervention effectiveness and are therefore highly valuable for inclusion in a scoping review. The addition of Lazard et al. (2025) and Leung et al. (2025) strengthens this evidence base by extending it to peer-sharing behavior and AI-generated campaign materials, respectively, and Leung et al. (2025) in particular illustrates that pre-registration, a priori power analysis, and manipulation checks are achievable within this literature. However, despite their methodological strengths, the reviewed studies also reveal important limitations – particularly related to blinding, treatment checks, confounding control, and transparency – which should be taken into account when interpreting their findings and assessing the robustness of the evidence base.
